# S100A6 binds to annexin 2 in pancreatic cancer cells and promotes pancreatic cancer cell motility

**DOI:** 10.1038/sj.bjc.6605289

**Published:** 2009-09-01

**Authors:** T Nedjadi, N Kitteringham, F Campbell, R E Jenkins, B K Park, P Navarro, F Ashcroft, A Tepikin, J P Neoptolemos, E Costello

**Affiliations:** 1 Division of Surgery and Oncology, University of Liverpool, Liverpool, UK; 2 Department of Pharmacology and Therapeutics, University of Liverpool, Liverpool, UK; 3 Department of Pathology, University of Liverpool, Liverpool, UK; 4 Cancer Research Programme, Municipal Institute of Medical Research, Barcelona, Spain; 5 Baylor College of Medicine, Houston, TX, USA; 6 Department of Physiology, University of Liverpool, Liverpool, UK

**Keywords:** pancreatic cancer, S100A6, annexin2, proteomics, immunoprecipitation

## Abstract

**Background:**

High levels of S100A6 have been associated with poor outcome in pancreatic cancer patients. The functional role of S100A6 is, however, poorly understood.

**Methods:**

Immunoprecipitation followed by two-dimensional gel electrophoresis and mass spectrometry were undertaken to identify S100A6 interacting proteins in pancreatic cancer cells. Immunohistochemistry and coimmunofluorescence were performed to examine expression or colocalisation of proteins. siRNA was used to deplete specific proteins and effects on motility were measured using Boyden Chamber and wound healing assays.

**Results:**

Our proteomic screen to identify S100A6 interacting proteins revealed annexin 11, annexin 2, tropomyosin *β* and a candidate novel interactor lamin B1. Of these, annexin 2 was considered particularly interesting, as, like S100A6, it is expressed early in the development of pancreatic cancer and overexpression occurs with high frequency in invasive cancer. Reciprocal immunoprecipitation confirmed the interaction between annexin 2 and S100A6 and the proteins colocalised, particularly in the plasma membrane of cultured pancreatic cancer cells and primary pancreatic tumour tissue. Analysis of primary pancreatic cancer specimens (*n*=55) revealed a strong association between high levels of cytoplasmic S100A6 and the presence of annexin 2 in the plasma membrane of cancer cells (*P*=0.009). Depletion of S100A6 was accompanied by diminished levels of membrane annexin 2 and caused a pronounced reduction in the motility of pancreatic cancer cells.

**Conclusion:**

These findings point towards a functional role for S100A6 that may help explain the link between S100A6 expression in pancreatic cancer and aggressive disease.

Pancreatic ductal adenocarcinoma is a leading cause of cancer-related deaths in the United States and Europe (Parkin *et al*, 2005; Jemal *et al*, 2008). The disease is characterised by rapid invasion which, combined with late diagnosis and ineffective treatment, leads to a dismal prognosis. The majority of patients die within 1 year of diagnosis (Ghaneh *et al*, 2007). A number of proteins of the S100 family, including S100A2, S100A4, S100A6, S100A11 and S100P have been shown to be overexpressed in pancreatic cancer (Crnogorac-Jurcevic *et al*, 2003; Shekouh *et al*, 2003; Vimalachandran *et al*, 2005; Ohuchida *et al*, 2006, 2007a). In many cases, overexpression has been associated with enhanced invasion (Ohuchida *et al*, 2005; Suemizu *et al*, 2007; Whiteman *et al*, 2007) or poorer outcome (Vimalachandran *et al*, 2005; Oida *et al*, 2006; Ohuchida *et al*, 2007a). Virtually all S100 proteins act as Ca^2+^ sensors. They participate in Ca^2+^ signal transduction by binding and modifying the activities of specific target proteins. They are expressed in a cell-specific manner, and they regulate a large variety of intracellular activities. Some S100 proteins are secreted and a number have been shown to bind the receptor for advanced glycation end products (Donato, 2007; Heizmann *et al*, 2007).

Several studies have identified overexpression of S100A6 in pancreatic cancer (Crnogorac-Jurcevic *et al*, 2003; Logsdon *et al*, 2003; Shekouh *et al*, 2003), in PanIN lesions (Vimalachandran *et al*, 2005) and in intraductal papillary mucinous neoplasms (Ohuchida *et al*, 2007b). Moreover, high level expression has been linked to poor outcome (Vimalachandran *et al*, 2005). A functional role for S100A6 in pancreatic cancer has, nevertheless, remained elusive. Ohuchida *et al* (2005) showed decreased invasion of pancreatic cancer cells after depletion of S100A6, although the means by which S100A6 influences invasion is unclear. To identify possible mechanisms underlying S100A6 activity, we undertook an unbiased proteomic screen for the detection of S100A6 interacting proteins in pancreatic cancer cells. This process involved a combination of immunoprecipitation, two-dimensional gel electrophoresis (2-DE) and mass spectrometry and revealed annexin 11, annexin 2, tropomyosin *β* as well as a novel candidate interactor, lamin B1. The functional roles of annexin 11 and lamin B1 are relatively poorly understood. By contrast, annexin 2 and tropomyosin have been much studied and have roles in cytoskeletal organisation and regulation of actin dynamics (Gerke *et al*, 2005; Gunning *et al*, 2008). We chose to focus further studies on annexin 2 as, like S100A6 (Vimalachandran *et al*, 2005) it is also overexpressed in preneoplastic pancreatic lesions (Sitek *et al*, 2005; Esposito *et al*, 2006) and is expressed in a similarly high proportion (>80%) of invasive cancers (Paciucci *et al*, 1998; Chen *et al*, 2005; Ortiz-Zapater *et al*, 2007). We report here colocalisation of annexin 2 and S100A6 in the plasma membrane of cultured pancreatic cancer cells and pancreatic tumours. Moreover, we report that membranous annexin 2 expression was more frequently observed in primary pancreatic tumours when cytoplasmic S100A6 levels were high (*P*=0.009) and that knockdown of S100A6 was accompanied by a reduction in cells expressing membranous annexin 2. These observations prompted us to investigate whether S100A6 contributed to motility in pancreatic cancer cells. We show here for the first time that depletion of S100A6 has a profoundly negative effect on the motility of pancreatic cancer cells. This finding may help explain the association of S100A6 with aggressive disease.

## Materials and methods

### Cell lines

The human pancreatic cancer cell lines Panc-1, MiaPaCa-2 and Suit-2 were maintained in RPMI 1640 medium supplemented with 10% fetal calf serum, 2 mM L-glutamine, 2500 iu ml^−1^ penicillin and 5 *μ*g ml^−1^ streptomycin (all from Sigma, Poole, UK) at 37°C in a humidified atmosphere of 5% CO_2._

### Immunoprecipitation

Panc-1 cells were washed twice with PBS, resuspended in SLIP buffer (50 mM Hepes, pH 7.5, 150 mM NaCl, 10% glycerol, 0.1% Triton X-100 and 0.5 mg ml^−1^ BSA) supplemented with protease inhibitors (Roche Diagnostics, Indianapolis, IN, USA) then sonicated for 15 s at 4°C. Lysates were clarified by centrifugation at 16 000 *g* for 10 min then the protein quantified using a BCA protein assay kit. For analysis of proteins on relatively large (18 × 13 cm format) 2-D gels, 8 mg protein lysate was precleared by incubating with Protein G-Sepharose beads (GE Healthcare Life Sciences, Buckinghamshire, UK) for 2 h at 4°C followed by centrifugation for 1 min at 16 000 *g*. Supernatants were then incubated with 4 *μ*g of anti-S100A6 antibody, 4 *μ*g antiannexin 2 antibody or 4 *μ*g of isotype control IgG or in the absence of any antibody (beads alone control) and gently mixed overnight at 4°C. Lysates were incubated with 100 *μ*l of prewashed Protein G-sepharose beads for a further 2 h with mixing. The immunoprecipitates were centrifuged, washed three times with SLIP buffer and resuspended in 50 *μ*l of 2-D lysis buffer (7 M urea, 2 M thiourea, 4% 3-[(3-cholamidopropyl) dimethylammonio]-1-propane sulphonate, 40 mM Tris base and 1% dithiothreitol). Samples were stored at −80°C until analysis by 1-D SDS–PAGE (using 15% Tris/Tricine gels) and western blotting or 2-D gel electrophoresis, as described below.

### Two-Dimensional Gel Electrophoresis and protein identification

Immunoprecipitated proteins were separated by 2-D gel electrophoresis as described earlier (Shekouh *et al*, 2003; Sheikh *et al*, 2007). Gel images were captured on a GS-800 gel scanner (BioRad, Hertfordshire, UK) and analysed using Progenesis software (NonLinear Dynamics, Newcastle upon Tyne, UK). Protein spots were excised, destained, trypsin digested and subjected to MALDI-TOF MS and/or LC-MS/MS as described earlier (Shekouh *et al*, 2003; Sheikh *et al*, 2007). Data were again submitted to Mascot and the NCBI database searched with the MS tolerance set to 1.2 Da and the MS/MS tolerance to 0.6 Da, with carbamidomethyl as a fixed modification.

### Small interference RNA-mediated knockdown of S100A6 and annexin 2

For small interference RNA (siRNA) treatment, cells were cultured in six-well plates at a density of 2 × 10^5^ cells per well (Panc-1 and Suit-2) or 4 × 10^5^ cells per well (MiaPaca-2). The medium was replaced 24 h later with antibiotic-free medium and cells were transfected with siRNA (10 nmol l^−1^) using Lipofectamine 2000 and Optimem I (both from Invitrogen, Carlsbad, CA, USA) according to the manufacturer's instructions. S100A6-targeting siRNA molecules: siRNA#1: 5′-AGA AGG AGC UCA CCA UUG G-3′, siRNA#2: 5′-UUG CAA GGC UGA UGG AAG A-3′, siRNA#3: 5′-ACA AGG ACC AGG AGG UGA A-3′, siRNA#4: 5′-GGG CCU UGG CUU UGA UCU AUU-3′, as well as an annexin 2-targeting siRNA: 5′-AGA CCA AAG GUG UGG AUG AUU-3′ were purchased from Dharmacon (Dharmacon Inc., Chicago, IL, USA). Three control siRNAs (10 nmol l^−1^) were used, including a ‘RISC-free’ siRNA (sicontrol RISC-free siRNA 1, Dharmacon Inc.) and two non-targeting siRNAs designated control 1 (sicontrol non-targeting siRNA 1 from Dharmacon) and control 2 (GGA CGC AUC CUU CUU AA, a gift from Dr M Boyd, University of Liverpool, Liverpool, UK). S100A6 levels diminished between 48 and 72 h after transfection with S100A6-targeting siRNAs (all three cell lines) and remained low out to 120 h (not shown).

### SDS–PAGE and western blotting analysis

Cell lysates were prepared by extraction into 100 mM Tris–HCl (pH 6.8) containing 2% w/v SDS and a protease inhibitor cocktail (Complete, Mini, EDTA-free protease inhibitors; Roche Applied Science, UK). Protein samples were quantified using a BCA protein assay kit (Perbio Science Ltd, Cramlington, UK) then subjected to SDS–PAGE (using 15% Tris/Tricine gels). Separated proteins were transferred to hybond nitrocellulose membranes (Amersham Biosciences, Bucks, UK). Membranes were then blocked for 1 h with PBS containing 0.1% Tween-20 (PBS-T) and 5% milk (Bio-Rad Laboratories Ltd., Hemel Hempstead, Hertfordshire, UK). Primary antibodies for detecting S100A6 (DakoCytomation, Cambridgeshire, UK), annexin-2 (Abcam plc, Cambridge, UK), and *β*-actin (clone AC-15, Sigma, Poole, UK) were detected by incubating membranes with horseradish-peroxidase (HRP)-conjugated secondary antibodies (Dako). Bound HRP was visualised using the enhanced chemiluminescence kit (PerkinElmer Life Sciences, Bucks, UK).

### Motility and invasion assays

Seventy-two hours after transfection with S100A6-targeting siRNA, annexin 2-targeting siRNA or control siRNA, pancreatic cancer cells were plated (5 × 10^4^ for Panc-1, 1 × 10^4^ for both MiaPaca-2 and Suit-2) for Boyden Chamber motility assays, conducted over 18 h, as described earlier (Thompson *et al*, 2007). Results are reported as motility indexes, which represent the number of cells translocating across the membranes after treatment with control- or S100A6/annexin 2-targeting siRNAs, expressed as a proportion of the number translocating in the RISC-free control. For invasion assays, Matrigel-coated Transwell plates (BD Biosciences, Oxford, UK) were used. Wound healing assays were performed as described earlier (Thompson *et al*, 2007) using cells harvested 72 h after treatment with siRNAs. Results were expressed as a migration index, that is, the distance migrated by siRNA treated (control or targeted) relative to the distance migrated by RISC-free control RNA-treated cells.

### Proliferation assays

The incorporation of [^3^H]-thymidine into pancreatic cancer cells was studied using methods described earlier (Varro *et al*, 2007). Seventy-two hours after transfection with S100A6-targeting and control siRNAs, cells (5 × 10^4^) were cultured in six-well plates in RPMI-1640 containing 10% FBS. Eighteen hours later, [^3^H]-thymidine (2 *μ*Ci ml^−1^; Sigma) was added and cells were incubated for a further 2 h and washed three times with 2 ml of ice cold PBS. Trichloroacetic acid (2 ml, 5% in PBS) was added and dishes were incubated at 4°C for 20 min. Cells were washed twice with 2 ml of ice-cold ethanol and incubated in 1 ml of 0.1 M NaOH for 60 min at 60°C. Solubilised material was recovered, 10 ml of scintillation fluid added, and the radioactivity incorporated determined using a scintillation counter (Packard 1500 TRI-CARB, Boston, MA, USA). Results were expressed as a proliferation index, that is, the [^3^H]-thymidine incorporated after siRNA treatment/[^3^H]-thymidine incorporated by RISC-free control RNA-treated cells. Pancreatic cancer cells (Panc-1, MiaPaca-2 and Suit-2) were plated onto 96-well plates at 5 × 10^3^ cells per well, 72 h after transfection with S100A6-targeting and control siRNAs. After 18 h of incubation at 37°C, MTT assays were performed as described earlier (Thompson *et al*, 2007).

### Immunofluorescence and immunocytochemistry

Panc-1 cells (subjected to siRNA treatment or not) were grown on glass coverslips in RPMI-1640 containing 10% fetal bovine serum. After 24 h of incubation at 37°C, cells were washed twice with PBS, fixed in 2% paraformaldehyde dissolved in PBS, then permeabilised using Triton X-100 (0.1% in PBS). For immunofluorescence, cells were incubated with a PBS solution containing 2% bovine serum albumin, then for 1 h at 4°C with either antibodies against rabbit polyclonal S100A6 (A5115; Dako, 1 : 400 dilution) or mouse monoclonal annexin 2 (ab8146; Abcam, 1 : 200 dilution). Cells were then washed with PBS and incubated with Cy3-conjugated mouse or rabbit IgG and FITC-conjugated rabbit or mouse IgG secondary antibodies for 1 h. DNA was stained with DAPI. Coverslips were mounted and examined using a Nikon fluorescence microscope. For immunocytochemistry, after fixation and permeabilisation, cells were incubated in methanol containing 3% H_2_O_2_ for 30 min at room temperature followed by 2% BSA in PBS for 1 h at 4°C and overnight incubation at 4°C with mouse monoclonal annexin 2 antibody (1 : 200 dilution in 1% BSA/PBS). After three washes using PBS, HRP-conjugated secondary antibody, was applied for 1 h at room temperature (Envision kit, DakoCytomation) followed by treatment with diaminobenzidine as chromogenic substrate for 5 min. Slides were counterstained with hematoxylin, dehydrated, and mounted with DPX mountant (VWR International, Poole, UK).

### Immunohistochemistry of pancreatic cancer tissue microarrays

Immunohistochemical detection of annexin 2 was undertaken, using a rabbit polyclonal antibody against annexin 2 (Aguilar *et al*, 2004), on a pancreatic cancer tissue microarray (TMA) containing matched duplicate non-malignant (normal ducts) and malignant (tumour) cores from 79 patients treated at the Royal Liverpool University Hospital, Liverpool, UK, between 1994 and 2003, as described earlier (Shekouh *et al*, 2003; Thompson *et al*, 2007). Scoring of the pancreatic cancer TMA was performed by a pancreatic specialist histopathologist (FC). The information recorded included the subcellular location of annexin 2 staining, the intensity of staining (graded 0=negative; 1=weak; 2=moderate; and 3=strong) and the extent of staining (percentage of cells showing positive immunoreactivity: 0 – 100% of cells). A score was assigned for each cellular compartment=the intensity of staining × the percentage of cells stained. To obtain associations between S100A6 and annexin 2 expression, data were cross-tabulated and Fisher's two-sided exact tests applied or Mann–Whitney *U*-test performed. All statistical analyses were carried out using Statview V.5.01. Results were considered significant for values of *P*<0.05. S100A6 immunostaining was undertaken earlier (Vimalachandran *et al*, 2005).

## Results

### Identification of S100A6-binding proteins

**Figure 1:**
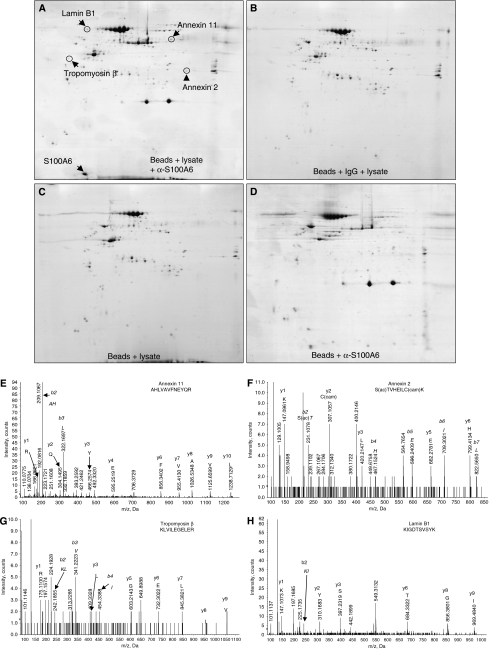
Identification of candidate S100A6-binding proteins by combined immunoprecipitation, 2-DE and MS. Two-dimensional gels display proteins precipitated after incubation of (**A**) Panc-1 cell lysate, S100A6 antibody and Protein G-sepharose beads (**B**) Panc-1 cell lysate, isotype antibody and Protein G-sepharose beads (**C**) Panc-1 cell lysate and Protein G-sepharose beads (**D**) Protein G-sepharose beads and S100A6 antibody. The protein spots circled in (**A**) were picked and proteins identified by MALDI-TOF MS and LC-MS/MS (**E**–**H**).

To identify potential S100A6-binding partners, we immunoprecipitated S100A6 from Panc-1 cell lysates and separated the recovered proteins by 2-DE. Protein spots observed on gels after immunoprecipitation with anti-S100A6 antibody (Figure 1A) but not on control gels (Figure 1B–D) were identified by MALDI-TOF mass spectrometry and confirmed by LC-MS/MS (Figure 1E–H; Supplementary Table 1). The identified proteins included the known binding partners annexin 11 (Mizutani *et al*, 1992), annexin 2 (Zeng *et al*, 1993), and tropomyosin (Golitsina *et al*, 1996) as well as a candidate novel interactor lamin B1. As both S100A6 and annexin 2 are overexpressed in pancreatic cancer, with expression occurring early in the development of the cancer in both cases (Sitek *et al*, 2005; Vimalachandran *et al*, 2005; Esposito *et al*, 2006; Ortiz-Zapater *et al*, 2007), we focused our attention particularly on annexin 2.

### Reciprocal immunoprecipitation of S100A6 by anti-annexin 2 and colocalisation of S100A6 and annexin 2

**Figure 2:**
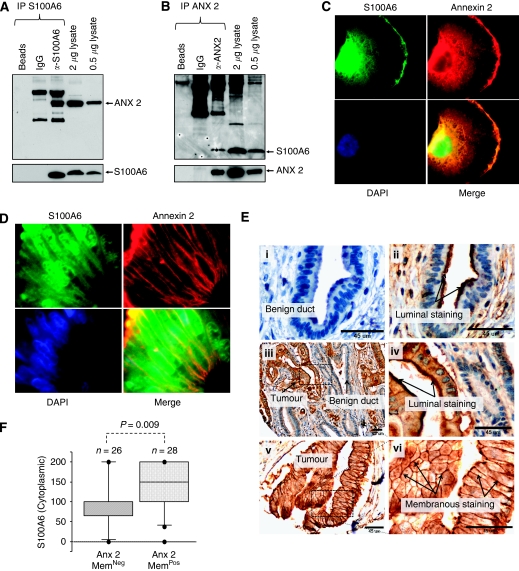
Association of S100A6 and annexin 2. (**A**) Proteins were precipitated from Panc-1 cell lysate using an S100A6 antibody (*α*-S100A6) or isotype control (IgG) or beads alone, as indicated, and subjected to immunoblotting analysis with annexin 2 (main panel) and S100A6 (lower panel) antibodies. The levels of annexin 2 and S100A6 detected in Panc-1 whole lysates (0.5 and 2.0 *μ*g) are also shown. (**B**) Recipricol-immunoprecipitation of proteins from Panc-1 cell lysate using an annexin 2 antibody (*α*–ANX-2), isotype control or beads alone followed by immunoblot (using 1 out of 5 of the recovered protein suspension) with S100A6 (main panel) or annexin 2 (lower panel) antibodies. (C and D) Immunofluorescence images of Panc-1 cells (**C**) and primary pancreatic tumour material (**D**) showing colocalisation, merged images (yellow), of S100A6 (green) and annexin 2 (red). Nuclei were labelled in blue using DAPI. Images were taken using 100 × objective. (**E**) Immunohistochemical staining of a pancreatic cancer tissue microarray using antiannexin 2 antibody. (i) Secondary antibody only; (ii) annexin 2 expression in non-neoplastic tissue showing apical membrane staining in a duct; (iii–vi) annexin 2 expression in malignant tissues showing apical membrane and membranous staining. Scale bars=45 *μ*m. (**F**) The staining scores for cytoplasmic S100A6 were plotted for primary tumours that were positive or negative for membranous annexin 2. The *P*-value shown is for comparison of the median scores of S100A6 for the positive and negative membranous annexin 2 staining, using the Mann–Whitney *U*-test.

The interaction between S100A6 and annexin 2 was further studied by immunoprecipitation followed by western analysis (Figure 2A and B). In keeping with the proteomic data (Figure 1), annexin-2 was readily detected (Figure 2A, main panel) by western blotting in complexes precipitated using an anti-S100A6 antibody (*α*-S100A6) from Panc-1 cell lysate. For reference, the detectable levels of annexin 2 in Panc-1 whole cell lysate are shown, as are the levels of S100A6 in the precipitated and whole cell lysate samples (Figure 2A, lower panel). Reciprocal immunoprecipitation with an annexin 2 antibody (*α*-ANX-2) followed by western analysis with an anti-S100A6 antibody revealed the specific detection of S100A6 in complexes precipitated from Panc-1 cell lysate (Figure 2B, main panel). The levels of annexin 2 in precipitated samples and, for reference, in whole cell lysates are shown (Figure 2B, lower panel). Coimmunoflourescence analyses of Panc-1 cells (Figure 2C) revealed colocalisation of S100A6 and annexin 2 in the cytoplasm with pronounced colocalisation in the plasma membranes. Colocalisation of S100A6 and Annexin 2 was also observed in the plasma membranes of paraffin-embedded sections from primary pancreatic tumour material (Figure 2D, see also Supplementary Figure 1).

### Membranous annexin 2 expression was more frequently observed in primary pancreatic tumours when cytoplasmic S100A6 levels were high

We described earlier the expression profile of S100A6 in tumours from 60 pancreatic cancer patients (Vimalachandran *et al*, 2005). To determine whether the overexpression of S100A6 occurred in tumours, which also overexpressed annexin 2, we investigated the expression of annexin 2 in this cohort (56 cases had sufficient material for evaluation). Immunohistochemical staining showed apical (luminal) membrane staining of annexin 2 in non-neoplastic ducts from all patients (*n*=25), where ducts were present on the TMA (Figure 2E ii). This was also observed in 78% of tumours (Figure 2E iii and iv), although less frequently in poorly differentiated tumours, in which only 60% (12 out of 20) of cases scored positively for apical membrane staining, compared with moderately and well-differentiated tumours where 92.5% (25 out of 27) and 88% (8 out of 9) of cases, respectively, were positive for this type of staining (*χ*^2^ *P*-value=0.01). Extensive annexin 2 staining was also detected in the cytoplasm of 76.3% of tumours (Figure 2E iii–vi). In 55% of tumours, distinct annexin 2 staining of the entire cell membrane was observed (Figure 2E v and vi). This type of staining was dubbed membranous staining. Eighty percentage of patients scored positively for both annexin 2 and for S100A6 (either cytoplasmic or nuclear), whereas 20% expressed either annexin 2 or S100A6 alone. Interestingly, we observed that patients who expressed membranous annexin 2 had significantly higher cytoplasmic S100A6 scores (Figure 2F, Mann–Whitney *U*-test; *P*=0.009. See also Supplementary Figure 2). Associations between the expression of cytoplasmic or nuclear S100A6 and apical membrane annexin 2 (Mann–Whitney *U*–test; *P*=0.38 and *P*=0.97, respectively) or cytoplasmic annexin 2 (Spearman Rank *ρ*=0.06, *P*=0.51; *ρ* −0.17, *P*=0.23, respectively) could not be established.

### RNAi-mediated depletion of S100A6 results in loss of annexin 2 from the plasma membrane

**Figure 3:**
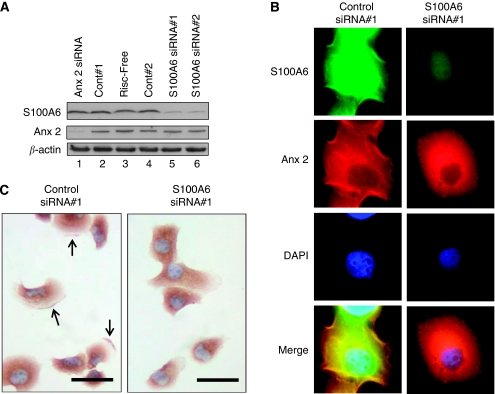
Knockdown of S100A6 was accompanied by loss of membranous annexin 2. (**A**) Western analysis of Panc-1 lysates 72 h after transfection with S100A6-targeted, annexin 2-targeted and control siRNAs. (**B**) Immunofluorescence of Panc-1 cells for the detection of S100A6 or annexin 2 performed 72 h after transfection with S100A6- or control siRNAs. Nuclei were stained with DAPI. (**C**) Immunocytochemistry of Panc-1 cells transfected with S100A6 siRNA#1 or control siRNA#1 and stained with antiannexin 2 antibody. Scale bars=50 *μ*m.

To further explore the relationship between the levels of S100A6 and membranous annexin 2, we examined whether diminishing the levels of S100A6 in a pancreatic cancer cell line would affect the overall levels or localisation of annexin 2. After siRNA-mediated depletion of S100A6 from Panc-1 cells, the overall levels of annexin 2 appeared unchanged (Figure 3A, lanes 5 and 6). Similarly, depleting annexin 2 did not appear to affect the protein levels of S100A6 (Figure 3A, lane 1). However, the levels of annexin 2 in the plasma membrane, which were detectable by immunofluoresence (Figure 3B) or immunocytochemistry (Figure 3C) were markedly reduced in S100A6-depleted cells. In control-siRNA-transfected cells, 37% of cells examined (*n*=1100; two independent experiments) were devoid of membranous annexin 2 staining. However, after S100A6 depletion, 59% of cells were found to lack membranous annexin 2 (*n*=862 cells; two independent experiments; *P*=0.01).

### RNAi-mediated reduction in S100A6 expression dramatically impaired cell motility

**Figure 4:**
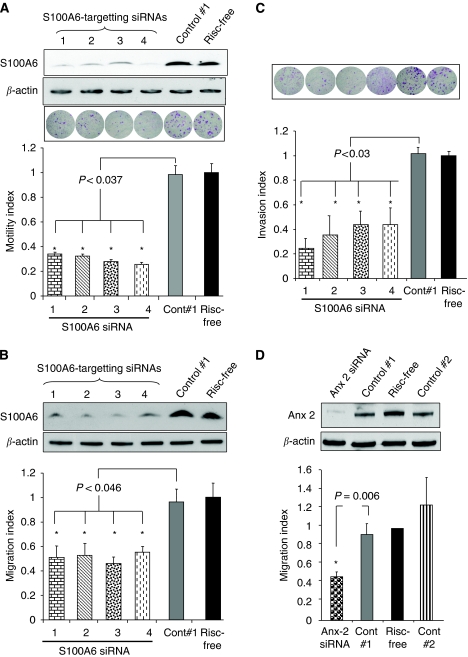
Expression of S100A6 and annexin 2 is associated with reduced pancreatic cancer cells motility. (**A**) Western analysis showing reduced S100A6 protein levels in Panc-1 cells 72 h after treatment with four different S100A6-targeting siRNAs and two control siRNAs. Motility was significantly reduced after S100A6 depletion compared with control-siRNA-transfected cells (*n*=5 experiments performed in triplicate). (**B**) Depletion of S100A6 expression inhibits wound healing. Western blot analysis of protein lysates from Panc-1 cells 72 h after transfection with four S100A6-targeting and control siRNAs. Histograms illustrate the motility index for wound healing assay using Panc-1 cell line. (**C**) Panc-1 invasiveness was significantly reduced in S100A6 siRNA-transfected cells compared with control-siRNA-transfected cells (*n*=6 experiments performed in triplicate). (**D**) Western analysis showing reduced annexin 2 protein levels in Panc-1 cells 72 h after treatment with targeting siRNAs and control siRNAs. Motility was significantly reduced in the annexin 2-targeting siRNA transfected Panc-1 cells compared with control-siRNA-transfected cells (*n*=3 experiments performed in triplicate). Error bars represent the standard error. The *P*-values were obtained using the Student's paired *t*-test, two-tailed.

Given the association of S100A6 with annexin 2 and the role of the latter in actin binding and motility, we questioned whether S100A6 may therefore contribute to the motility of pancreatic cancer cells. Transfection of Panc-1 cells with four S100A6-targeting siRNAs effectively reduced S100A6 protein levels (Figure 4A) and caused a significant reduction in motility, from an average of 65.4% (S100A6 siRNA#1) to 73.8% (S100A6 siRNA#4) compared with a non-targeting control siRNA (*P*<0.037). Similarly, transfection of MiaPaca-2 and Suit-2 cells with S100A6 siRNA#1 caused depletion of S100A6 and was accompanied by an average decrease of 73.4 and 64.2% in motility compared with the non-targeting control siRNA (*P*=0.003 and *P*=0.03, respectively; Supplementary Figure 3A and B). Wound healing assays were also undertaken. Depletion of S100A6 from Panc-1 cells, using four independent S100A6-targeting siRNAs (Figure 4B), reduced wound healing by at least 42% in each case compared with control-siRNA-treated cells (*P*=0.03, siRNA#1; *P*=0.02, siRNA#2; *P*=0.02, siRNA#3; *P*=0.05, siRNA#4). In a similar manner, depletion of S100A6 from Suit-2 cells using four S100A6-targeting siRNAs (Supplementary Figure 3C) significantly reduced wound closure (*P*=0.002, siRNA#1, *P*=0.05, siRNA#2, *P*=0.01, siRNA#3, *P*=0.02, siRNA#4) by averages of 30% (S100A6 siRNAs#2) and 58.5% (S100A6 siRNAs#1). We also confirmed that depletion of S100A6 was associated with a significant reduction in invasion through matrigel-coated microporous membranes (Figure 4C), consistent with the report by Ohuchida *et al* (2005). Of note, the effects of annexin 2 on pancreatic cancer cell motility have never been reported. We observed that transfection of Panc-1 cells with an annexin 2-targeting siRNA (Figure 4D) was accompanied by a 50% decrease in the cell motility index compared with control siRNA#1 as assessed by modified Boyden chamber assay. We noted that 72 h after transfection with the annexin 2-targeting siRNA, there was also a 36% decrease in viable Panc-1 cell number, compared with control siRNA#1 (*n*=5 independent experiments, *P*=0.001).

### Depletion of S100A6 did not affect cell proliferation

**Figure 5:**
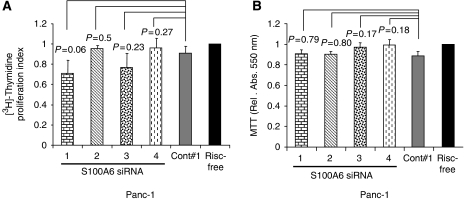
Determination of the effects of S100A6 knockdown on cell proliferation. (**A**) [^3^H]-thymidine incorporation was performed 72 h after transfection with 10 nm S100A6-targeting and control siRNAs. Histogram showing [^3^H]-thymidine incorporation in S100A6 depleted compared with control-siRNAs-treated Panc-1 cell line. Error bars represent standard error for five experiments performed in triplicate. (**B**) Panc-1 cells were plated 72 h after transfection with S100A6-targeting and control siRNAs and 18 h later MTT assays were performed. Results were expressed as MTT reading after siRNA treatment/MTT reading after RISC-free treatment. Error bars represent standard error for five experiments performed in triplicate.

To determine whether the reductions in cancer motility after depletion of S100A6 expression might reflect losses in growth, we undertook measurements of proliferation of cells 72 h after transfection with control- and S100A6-targeting siRNAs. The level of [^3^H]-thymidine incorporated into siRNA-treated cells (control or targeted) was expressed as a proportion of that incorporated in RISC-free control-treated cells. Depletion of S100A6 from Panc-1 cells did not lead to significant reductions in [^3^H]-thymidine incorporation compared with control-siRNA-treated cells (Figure 5A; *P*=0.06, siRNA#1; *P*=0.50, siRNA#2; *P*=0.23, siRNA#3; *P*=0.27, siRNA#4). We also examined the effect of depletion of S100A6 on cell proliferation by MTT assay. No significant changes in MTT readings were observed after depletion of S100A6 in Panc-1 cells (Figure 5B). Similarly, depletion of S100A6 in MiaPaca-2 and Suit-2 cells was not accompanied by any significant reductions in [^3^H]-thymidine incorporation (Supplementary Figure 4A and B; *P*=0.33, both cell lines) nor in MTT readings (Supplementary Figure 4C and D; *P*=0.32 and *P*=76 for MiaPaca-2 and Suit-2, respectively).

## Discussion

Although S100A6 overexpression in pancreatic and other cancers, including gastric (Yang *et al*, 2007), thyroid (Brown *et al*, 2006), breast (Cross *et al*, 2005) and colorectal (Komatsu *et al*, 2000) has been documented, its precise role in cancer is not known. In pancreatic cancer, the expression of S100A6 appears to correlate with aggressive disease in that high levels of tumour S100A6 are associated with poorer outcome (Vimalachandran *et al*, 2005), whereas pancreatic cancer cells depleted of S100A6 are less invasive (Ohuchida *et al*, 2005). A link between S100A6 and colon cancer invasion has also been suggested by the observation that increased expression of S100A6 is found at the leading edges of colon cancer tissues (Komatsu *et al*, 2000). However, the molecular mechanism responsible for these effects has not been characterised. Our proteomic-based approach to identify S100A6-binding partners was performed with the aim of shedding light on S100A6 function in pancreatic cancer.

Our study identified the known S100A6-binding proteins, tropomyosin *β*, annexin 11 and annexin 2 as well as novel binding protein lamin B1, as candidate S100A6 interactors in pancreatic cancer cells. The interaction and the colocalisation of S100A6 and tropomyosin have been documented (Golitsina *et al*, 1996; Orre *et al*, 2007). Tropomyosins have been shown to regulate microfilament organisation and actin dynamics (Boyd *et al*, 1995; Gunning *et al*, 2008), with reduction in tropomyosin 1 levels leading to anchorage independent growth (Boyd *et al*, 1995). Breen *et al* observed disorganisation of tropomyosin associated cytoskeleton filament networks after depletion of S100A6 from fibroblasts (Breen and Tang, 2003). Although we did not study the interaction between tropomyosin and S100A6 here, it is possible that the loss of motility observed after S100A6 depletion is caused, at least in part, by an involvement of S100A6 with tropomyosin. Annexin 11 is widely expressed and roles in cell division (Farnaes and Ditzel, 2003) and cytokenesis (Tomas *et al*, 2004) have been reported. The binding of annexin 11 to S100A6 has been shown to take place in the nuclear envelope (Tomas and Moss, 2003), where the complex may have a function in membrane deconstruction during nuclear envelope breakdown in mitosis. Lamin B1 is a major component of the nuclear lamina that influences many aspects of cellular functions including proliferation, differentiation, transcription and apoptosis (Dechat *et al*, 2008). Although it has not been reported to bind S100A6, lamin B has been shown to colocalise with S100A6 at focal points within the nucleus (Tomas and Moss, 2003).

Annexin 2 is a member of a family of calcium-dependent, phospholipid-binding proteins, possessing a number of intracellular activities such as regulation of exocytosis, and extracellular activities such as fibrinolysis through its interaction with tissue plasminogen activator (tPA) (Gerke *et al*, 2005). It is present in most cells as either a monomeric form within the cytoplasm or as a tetramer, comprising two molecules of annexin 2 and two of S100A10, localised to the plasma membrane-actin cytoskeleton interface. Although S100A10 is the best-characterised binding partner, annexin 2 has also been shown to interact with other S100 family members. For example, an interaction with S100A4 has been described (Semov *et al*, 2005). This interaction was accompanied by accelerated tPA-mediated plasminogen activation in solution as well as on endothelial cell surfaces (Semov *et al*, 2005), similar to that described for the Anx2/S100A10 complex. Interaction with S100A6 has also been reported (Zeng *et al*, 1993). Using matrix-immobilised S100A6, Zeng *et al* (1993) recovered annexin 2 from a preparation of bovine heart proteins. Our demonstration that S100A6 and annexin 2 coimmunoprecipitate with either S100A6 or annexin 2 antibodies, along with their colocalisation within the cytoplasm and membranes of pancreatic cancer cells, suggest that S100A6 may be a significant annexin 2-binding partner in pancreatic cancer cells. Moreover, our observation that high levels of S100A6 correlated with the expression of membranous annexin 2 in patient tumours whilst low S100A6 levels correlated with a lack of membranous annexin 2 suggests that S100A6 may affect the localisation of annexin 2 to membranes. This was further supported by the finding that depletion of S100A6 in cultured pancreatic cancer cells was found to result in diminished numbers of cells expressing membranous annexin 2. A similar relationship between annexin 2 and S100A10 was reported by Deora *et al* (2004) who studied the translocation of annexin 2 from the cytoplasm to the cell surface of endothelial cells after the application of mild heat. The authors found that depletion of S100A10 from endothelial cells resulted in reduced surface expression of annexin 2, indicating that S100A10 expression was important for cell surface translocation of annexin 2.

The most striking phenotype of S100A6 depletion from pancreatic cancer cells in our study was the dramatic loss of cell motility, observed in both Boyden chamber and wound healing assays. This loss of motility is likely to contribute to the reduced invasion observed when S100A6 levels are depleted. Relatively little is known about the contribution of S100A6 to cellular motility. The only other published study, which to our knowledge examined the effect of S100A6 on motility, showed a decrease in the motility of one of three osteosarcoma cell lines after adenoviral-mediated overexpression of S100A6 (Luu *et al*, 2005). Interestingly in osteosarcoma, a trend was observed between decreased metastasis and increased S100A6 staining, contrasting the findings in melanoma and colorectal cancer (Maelandsmo *et al*, 1997; Komatsu *et al*, 2000). Whether S100A6 contributes to pancreatic cancer cell motility through interaction with annexin 2 is not yet clear. However, the observed positive correlation in this study between high S100A6 levels and the localisation of annexin 2 to the plasma membrane in pancreatic cancer cells may have significance with respect to the ability of these cells to move, as annexin 2 has been implicated in the regulation of actin dynamics, cell spreading and wound closure (Hayes *et al*, 2006; Babbin *et al*, 2007; de Graauw *et al*, 2008). Hayes *et al* showed that annexin 2 was concentrated in the dynamic actin-rich protrusions of motile cells and that siRNA-mediated depletion of annexin 2 led to loss of protrusive and retractile activity (Hayes *et al*, 2006). More recently, Babbin *et al* (2007) observed that depletion of annexin 2 from Caco-2 epithelial cells resulted in reductions in cell spreading and wound closure. Finally, the work of de Graauw *et al* (2008) pointed towards the phosphorylation of annexin 2 as a key event in the remodelling of the actin cytoskeleton during cell spreading. Thus, if high levels of S100A6 promote or facilitate annexin 2 translocation to the cell membrane, this may positively enhance cell motility. However, we cannot rule out the possibility that the relationship between membranous expression of annexin 2 and S100A6 level is a consequence, rather than a cause, of the effect of S100A6 on motility. In addition, the interaction between annexin 2 and S100A6 may contribute to increased tPA activity, which could lead to a variety of effects, potentially including increased cell motility (Ortiz-Zapater *et al*, 2007; Sharma and Sharma, 2007).

In summary, our study has provided insight into candidate S100A6-binding partners in pancreatic cancer and has shown a positive relationship between the cellular levels of S100A6 and the localisation of annexin 2 to the cell membrane. Finally, our findings provide new insight into S100A6 function, namely that it promotes pancreatic cancer cell motility.
