# Supplementary material for: S100A6 binds to annexin 2 in pancreatic cancer cells and promotes pancreatic cancer cell motility
Source: Br J Cancer. 2009 Sep 1;101(7):1145–54. doi: 10.1038/sj.bjc.6605289 (PMC2768105; doi:10.1038/sj.bjc.6605289)
Supplement: Supplementary Figure 1 [file 6605289x1.ppt]

## Slide 1
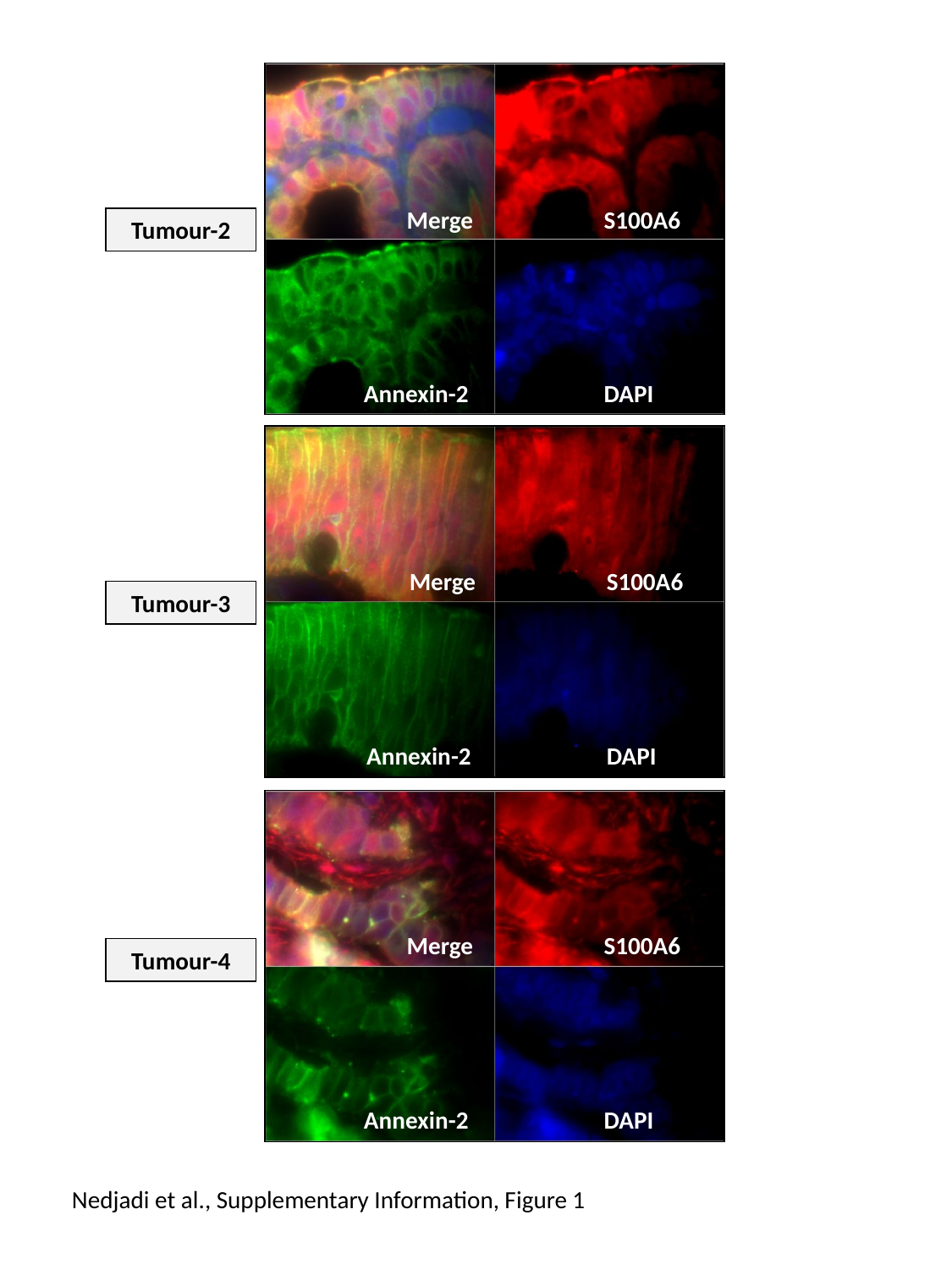

Merge
S100A6
Annexin-2
DAPI
Tumour-2
Merge
S100A6
Annexin-2
DAPI
Tumour-3
Merge
S100A6
Annexin-2
DAPI
Tumour-4
Nedjadi et al., Supplementary Information, Figure 1
