# Supplementary figures and images for: S100A6 binds to annexin 2 in pancreatic cancer cells and promotes pancreatic cancer cell motility
Source: Br J Cancer. 2009 Sep 1;101(7):1145–54. doi: 10.1038/sj.bjc.6605289 (PMC2768105; doi:10.1038/sj.bjc.6605289)

## Slide 1
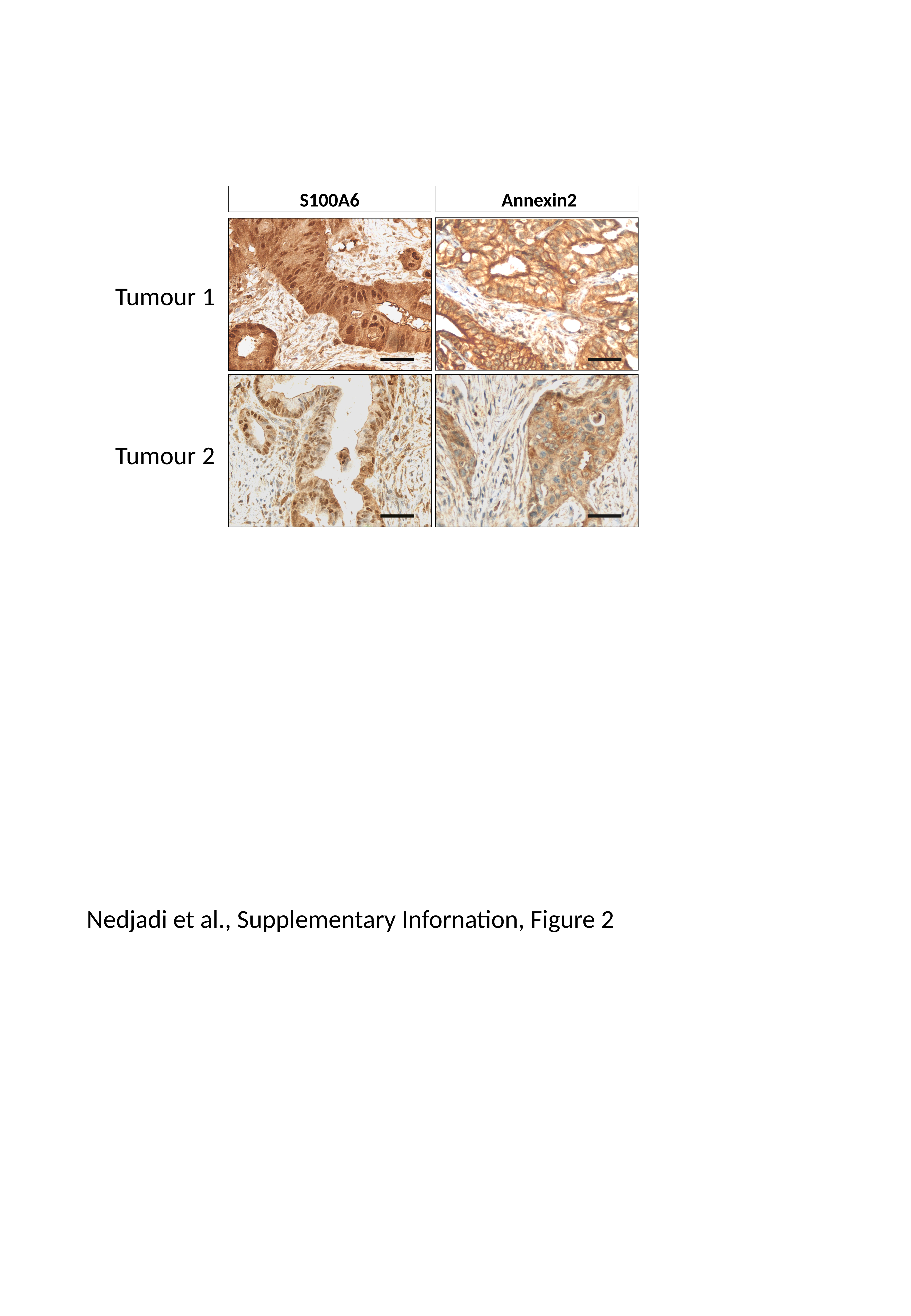

S100A6
 Annexin2
Tumour 1
Tumour 2
Nedjadi et al., Supplementary Infornation, Figure 2

Supplement: Supplementary Figure 2 [file 6605289x2.ppt]
