# Supplementary material for: S100A6 binds to annexin 2 in pancreatic cancer cells and promotes pancreatic cancer cell motility
Source: Br J Cancer. 2009 Sep 1;101(7):1145–54. doi: 10.1038/sj.bjc.6605289 (PMC2768105; doi:10.1038/sj.bjc.6605289)
Supplement: Supplementary Figure 3 [file 6605289x3.ppt]

## Slide 1
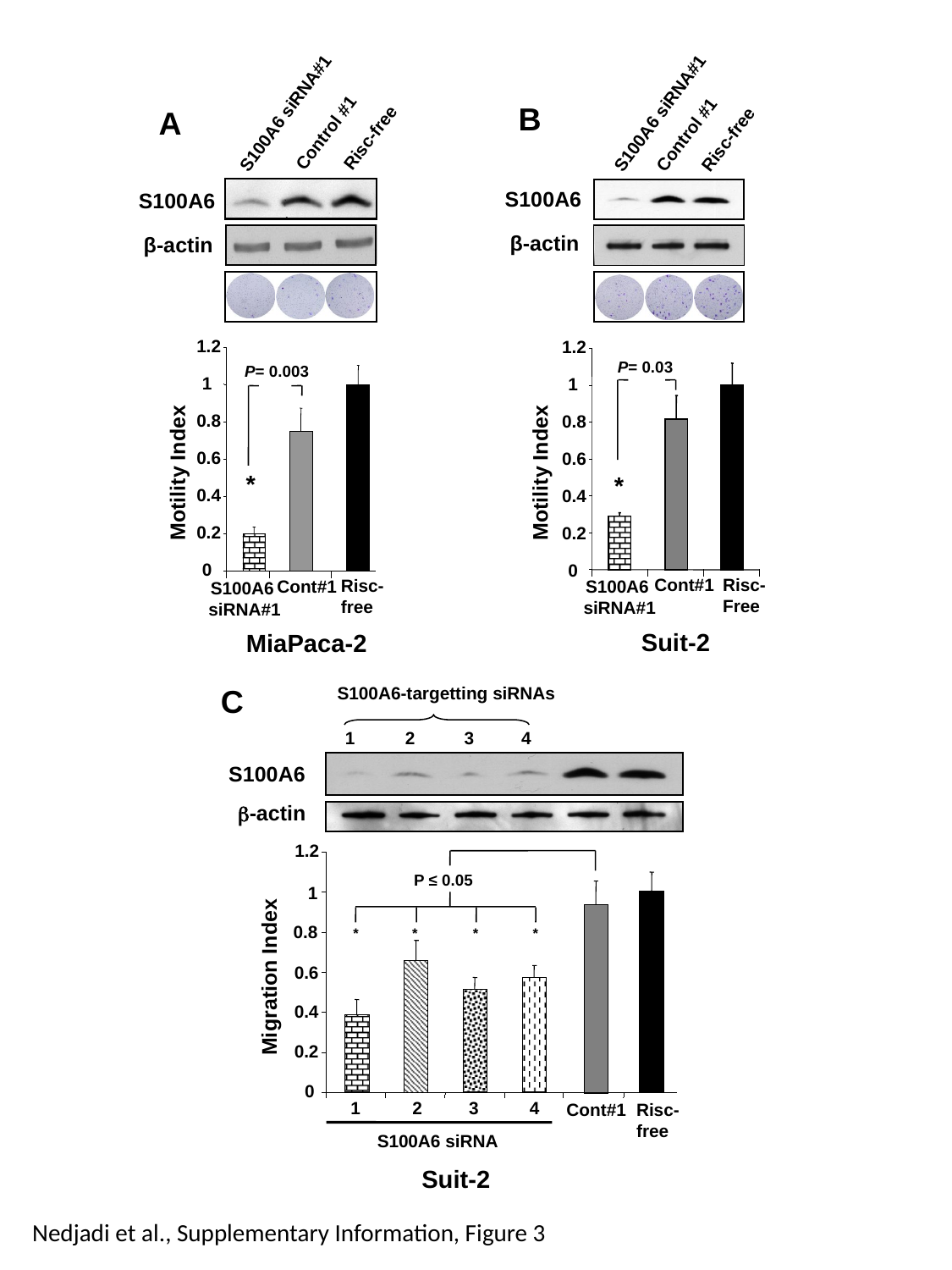

S100A6 siRNA#1
S100A6 siRNA#1
B
Risc-free
Risc-free
A
Control #1
Control #1
S100A6
S100A6
β-actin
β-actin
1.2
1.2
P= 0.03
P= 0.003
1
1
0.8
0.8
Motility Index
Motility Index
0.6
0.6
*
*
0.4
0.4
0.2
0.2
0
0
Cont#1
Risc-
Free
S100A6
 siRNA#1
Risc-
free
Cont#1
S100A6
 siRNA#1
Suit-2
MiaPaca-2
C
S100A6-targetting siRNAs
1
2
3
4
S100A6
β-actin
1.2
1
0.8
0.6
Migration Index
0.4
0.2
0
P ≤ 0.05
*
*
*
*
1
2
3
4
Cont#1
Risc-free
S100A6 siRNA
Suit-2
Nedjadi et al., Supplementary Information, Figure 3
