# Supplementary material for: S100A6 binds to annexin 2 in pancreatic cancer cells and promotes pancreatic cancer cell motility
Source: Br J Cancer. 2009 Sep 1;101(7):1145–54. doi: 10.1038/sj.bjc.6605289 (PMC2768105; doi:10.1038/sj.bjc.6605289)
Supplement: Supplementary Figure 4 [file 6605289x4.ppt]

## Slide 1
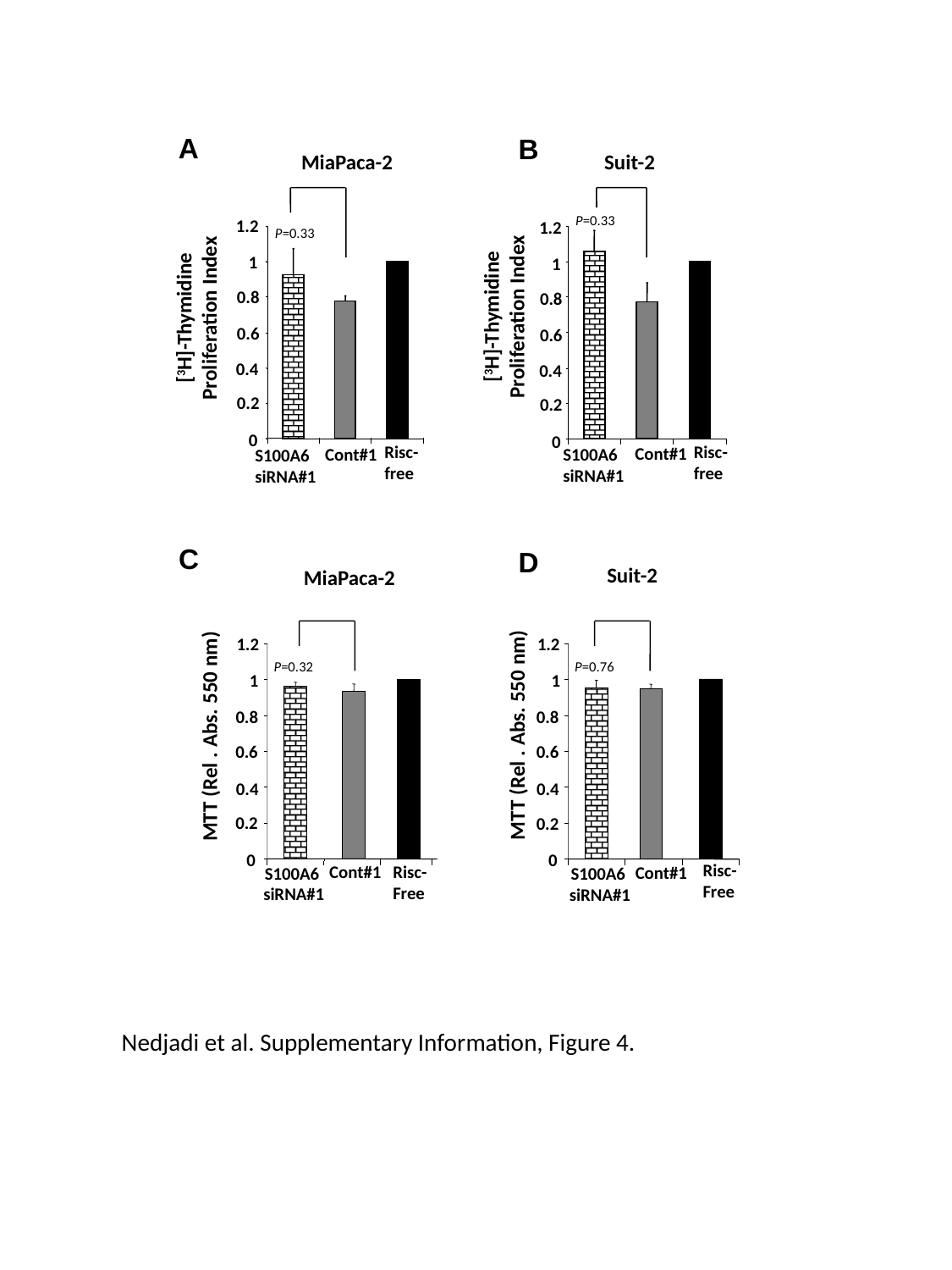

A
B
MiaPaca-2
Suit-2
P=0.33
1.2
1.2
1
0.8
0.6
0.4
0.2
0
P=0.33
1
[3H]-Thymidine Proliferation Index
[3H]-Thymidine Proliferation Index
0.8
0.6
0.4
0.2
0
Risc-free
Risc-free
Cont#1
Cont#1
S100A6 siRNA#1
S100A6 siRNA#1
C
D
Suit-2
MiaPaca-2
1.2
1
0.8
0.6
0.4
0.2
0
1.2
1
0.8
0.6
0.4
0.2
0
P=0.76
P=0.32
MTT (Rel . Abs. 550 nm)
MTT (Rel . Abs. 550 nm)
Risc-
Free
Cont#1
Risc-
Free
Cont#1
S100A6
 siRNA#1
S100A6
 siRNA#1
Nedjadi et al. Supplementary Information, Figure 4.
