# Supplementary material for: S100A6 binds to annexin 2 in pancreatic cancer cells and promotes pancreatic cancer cell motility
Source: Br J Cancer. 2009 Sep 1;101(7):1145–54. doi: 10.1038/sj.bjc.6605289 (PMC2768105; doi:10.1038/sj.bjc.6605289)
Supplement: Supplementary Information [file 6605289x5.doc]

**Supplementary Information: Figures Legends**

**Supplementary Figure 1.** Immunofluorescence images of primary pancreatic tumour material (tumours 2-4) showing co-localisation, merged images (yellow), of S100A6 (red) and annexin 2 (green). Nuclei were labeled in blue using DAPI. Images were taken using 100 X objective.

**Supplementary Figure 2.** Shown is an example of a tumour (Tumour 1) that expresses high levels of cytoplasmic S100A6 and has intense annexin 2 staining at the plasma membranes. Tumour 2 is an example of a tumour that contains low cytoplasmic S100A6 and lacks detectable annexin 2 staining at the plasma membranes. Scale bars = 50 µm.

**Supplementary Figure 3.** Knockdown of S100A6 is associated with decreased motility of pancreatic cancer cells. Western analysis demonstrating reduced S100A6 protein levels in MiaPaca-2 (A) and Suit-2 (B) cells 72 h post-treatment with S100A6-targeting siRNA#1 and two control-siRNAs. Motility (as measured by modified Boyden Chamber assay) was significantly reduced in S100A6-targeting siRNA transfected cells compared to control-siRNA transfected cells, in both cell lines (n = 5 experiments performed in triplicate, error bars represent the standard error). (C) Depletion of S100A6 expression inhibits wound healing. S100A6 silencing was assessed by Western blot analysis of protein lysates from Suit-2 cells 72h post-transfection with four different S100A6-targeting and control siRNAs. Histograms illustrate the motility index for wound healing assay (n=6 experiments performed in triplicate, error bars represent the standard error). The *P* values were obtained using the Student’s paired t-test, two-tailed.

**Supplementary Figure 4.** Determination of the effects of S100A6 knock-down on cell proliferation. (**A**) and (B) Histograms showing [^3^H]-thymidine incorporation in S100A6-depleted compared to control siRNAs-treated MiaPaca-2 (A) and Suit-2 (B) cell lines. (C) and (D) MTT assays were performed 72 h post-transfection with S100A6-targeting and control- siRNAs using MiaPaca-2 (A) and Suit-2 (B) cell lines. Results were expressed as MTT reading following siRNA treatment/MTT reading following RISC-free treatment. Error bars represent standard error for five experiments carried performed triplicate.
